# Whole genome and phylogenomic insights into *Vibrio parahaemolyticus* from Pacific White Shrimp reveal resistance and virulence traits in Bangladeshi aquaculture

**DOI:** 10.1371/journal.pone.0346962

**Published:** 2026-04-09

**Authors:** Md. Habib Ullah Masum, Mohammad Sharif Uddin, Kazi Chamonara, Sumaia Sultana, Sajedul Islam, Afifa Siddiqua

**Affiliations:** 1 Department of Genomics and Bioinformatics, Faculty of Biotechnology and Genetic Engineering, Chattogram Veterinary and Animal Sciences University (CVASU), Khulshi, Chattogram, Bangladesh; 2 Department of Microbiology, Noakhali Science and Technology University (NSTU), Noakhali, Bangladesh; 3 Department of Environmental Biotechnology, Faculty of Biotechnology and Genetic Engineering, Chattogram Veterinary and Animal Sciences University (CVASU), Khulshi, Chattogram, Bangladesh; 4 Department of Physiology, Biochemistry and Pharmacology, Chattogram Veterinary and Animal Sciences University (CVASU), Khulshi, Chattogram, Bangladesh; Defense Threat Reduction Agency, UNITED STATES OF AMERICA

## Abstract

The fisheries sector of Bangladesh has significantly contributed to the national economy and provided livelihoods for millions of rural households. However, disease outbreaks and contamination of fish by *Vibrio* species pose significant hurdles to production and trade. This study analyzed the whole genomes of *V. parahaemolyticus* (SU37A and SU91A) isolates from Pacific white shrimp using the Nanopore sequencing platform. Subsequent *de novo* genome assemblies of the strains revealed high-quality, genome profiles. Further annotation identified multiple antimicrobial resistance genes (*vanT, vanY, tet, adeF*, and *CARB-18*) in the strains, conferring resistance to various antibiotic classes. Mobile genetic elements were located adjacent to key regulatory genes associated with antibiotic resistance, indicating potential acquisition and dissemination of resistance phenotypes among the strains. Most of the identified virulence-associated genes are related to regulatory and stress response functions (*rpoS*, *rpoE*, *crp*, *hfq*, *arcA*), which facilitate adaptation to nutritional scarcity, oxidative stress, and host immune responses. Functional subsystem analysis demonstrated a high degree of metabolic adaptability. Phylogenomic analysis showed that the SU91A clustered with the clinical strain 3148−98, while the SU37A aligned with the non-clinical strain 11−2, suggesting potential zoonotic characteristics. These findings contribute to understanding the pathophysiology of this opportunistic bacterium during infection and elucidate the molecular factors that enable *V. parahaemolyticus* to adapt and persist in diverse environments.

## Introduction

Bangladesh ranks among the largest fish-producing nations, with a total harvest of approximately 5 million metric tons in the fiscal year 2023−24 [1,2]. The country has the third-highest inland open-water capture rate and the fifth-highest aquaculture production globally [1]. The aquaculture sector has become an inevitable source of food production, supporting farmers and rural households while contributing to national economic growth [1,3]. Fisheries, aquaculture, and related processing account for approximately 60% of the nation’s animal protein supply, underscoring their essential role in food security. These sectors also make substantial contributions to the national GDP and export revenues. Specifically, the fisheries sector contributed 2.53% to the national GDP, 22.26% to the agricultural GDP, and 0.90% to national export revenues in the fiscal year 2023–24. Although the fisheries sector’s share of GDP declined from 2.7% in 2019 to 2.41% in 2023, this decrease was primarily due to increased production costs, disease outbreaks, and other environmental hurdles [1]. *Vibrio* outbreaks have a significant socio-economic impact in Bangladesh, where shrimp exports contribute millions of USD annually and support employment for millions of rural workers [1,4]. Bacterial outbreaks reduce export competitiveness in stringent international markets and cause direct economic losses due to shrimp mortality [3]. Furthermore, contaminated shrimp have been linked to human foodborne diseases [5]. Such contamination frequently arises from inadequate hygienic practices during processing, preservation, and storage [6,7].

*Vibrio* infection in shrimp, which are natural inhabitants of estuarine and coastal ecosystems, poses a significant threat to aquaculture sustainability and public health in Bangladesh. Among the various bacterial pathogens present in seafood, such as shrimp, *Vibrio* species are most frequently associated with human diseases [7]. Notably, *Vibrio parahaemolyticus* can cause gastroenteritis and food poisoning when contaminated raw shrimp are consumed [8]. Consequently, shrimp contamination has emerged as a major concern for the sustainability of aquaculture and public health, both in Bangladesh and globally. Virulent *V. parahaemolyticus* strains produce several virulence factors during pathogenesis. Pathogenicity is primarily mediated by specific genetic elements, including the Type III Secretion System (T3SS), the Type VI Secretion System (T6SS), and regulatory genes such as *rpoE*, *rpoS*, *hfq*, and *fur*. These elements control the production of effector proteins and toxins, influencing cytotoxicity, intestinal colonization, and evasion of host immune responses [9,10]. Additional genes, such as *ascV*, a structural component of T3SS, directly facilitate the translocation of virulence factors into host cells [11]. The *tdh* (thermostable direct hemolysin) and *trh* (thermostable related hemolysin) genes serve as principal virulence markers of *Vibrio parahaemolyticus*. The *tdh* gene encodes a pore-forming hemolysin that damages intestinal epithelial cells and disrupts ion homeostasis, resulting in fluid secretion and gastroenteritis [12]. On the other hand, the *trh* gene encodes a hemolysin that is structurally and functionally related to thermostable direct hemolysin and has been associated with enterotoxic activity. Therefore, strains carrying either *tdh* or *trh* are considered potentially pathogenic, indicating a possible role in human infection [12,13]. Health risks are further compounded by the transmission of antibiotic resistance genes. Intensive shrimp farming practices often involve the use of antibiotics, including sulfonamides, fluoroquinolones, and tetracyclines, which can promote the emergence of antibiotic-resistant *Vibrio* strains in farmed shrimp [14–17]. The overuse of antibiotics in aquaculture accelerates the development of antibiotic resistance, thereby threatening public health and adversely affecting food production and healthcare systems. Even with increasing resistance, there is currently no FDA-approved method available for managing *Vibrio* infections in shrimp [18].

Whole-genome analyses of *V. parahaemolyticus* strains worldwide have demonstrated substantial genomic diversity in both virulence factors and host specificity [19]. Numerous virulence attributes are acquired via horizontal gene transfer (HGT) mediated by mobile genetic elements (MGEs) [20]. The genome plasticity and genetic diversity of MGEs associated with *V. parahaemolyticus* may influence the pathogen’s survival and infection potential. Furthermore, elucidating the relationship between epidemiological connections among pathogenic strains and genome complexity is essential [21,22]. Recent studies indicate that environmental isolates of *V. parahaemolyticus* can acquire virulence traits, rendering them potentially pathogenic to both humans and aquatic animals [22–26].

This study conducted whole-genome sequencing of *V. parahaemolyticus* isolated from Pacific white shrimp obtained at a fisheries market in Noakhali, Bangladesh. We hypothesized that shrimp-associated strains harbor distinct virulence determinants and accessory genomic elements enhancing pathogenicity and environmental adaptability, and evaluated this through pan-genome analysis to assess genomic diversity and identify candidate virulence-related features linked to persistence, growth, and opportunistic infection.

## Methods

### Ethical statement

The Ethics Approval Committee of Noakhali Science and Technology University (NSTUEC). reviewed and authorized the study protocol (Approval no. NSTU/SCI/EC/2025/451). All procedures complied with relevant regulations and legislation. The research site was neither privately owned nor protected, and the study was conducted in an area without endangered or protected species.

### Sample collection, processing and antibiotic resistance profiling

Pacific white shrimp samples were collected from four retail markets in Noakhali district, Bangladesh, in July 2024. These samples were collected aseptically in sterile, resealable containers. To prevent contamination, each shrimp sample was placed in a sterile plastic zipper container. The samples were transported on ice in a cooler box (maintained at a temperature below 4°C) from the sampling site to the Department of Microbiology at Noakhali Science and Technology University (NSTU) and were processed within a few hours. Shrimp samples were homogenized and enriched in alkaline peptone (APW; 3% NaCl, pH 8.5 ± 0.1) water at 37°C. Enriched cultures were inoculated onto thiosulfate–citrate–bile salts–sucrose (TCBS) agar plates and incubated for 18–24 hours at 37°C [27]. Suspected *Vibrio* isolates were identified using direct microscopy and a series of biochemical assays, including oxidase, catalase, methyl red, Voges-Proskauer, and urease tests [28,29]. A final confirmation step involved PCR detection of the genus-specific *groEL* gene. Amplification was performed using the primer pair groVp1 (5′-GTCAGGCTAAGCGCGTAAGCA-3′) and groVp2 (5′-GCATGCCTGCGCTTTCTTTTTG-3′) under primer-specific PCR conditions. The protocol included an initial denaturation at 94°C for 5 minutes, followed by 30 cycles of denaturation at 94°C for 30 seconds, annealing at 69°C for 30 seconds, and extension at 72°C for 30 seconds. A final extension was carried out at 72°C for 5 minutes [30]. Antibiotic resistance of the isolates were assessed using standardized protocols from the Clinical and Laboratory Standards Institute (CLSI, M45) [31], European Committee on Antimicrobial Susceptibility Testing (EUCAST, v.16.0) [32], and Clinical and Laboratory Standards Institute (CLSI, M100) [33]. Susceptibility profiles were determined for fourteen antibiotics (S1 Table). Multidrug-resistant (MDR) isolates were defined as those exhibiting resistance to at least one agent in three or more antimicrobial categories [34]. *V. parahaemolyticus* ATCC 17802 and *E. coli* ATCC 25922 were used as the positive and negative controls, respectively, in both assays. The experimental *V. parahaemolyticus* isolates were then selected based on their MDR profiles and subsequently subjected to whole genome sequencing (WGS).

### Whole genome sequencing, assembly and strain characterization

The total DNA from the isolates was extracted utilizing the QIAamp® DNA Mini Kit (QIAGEN, Germany) and then quantified with a Qubit 4 fluorometer (Thermo Fisher Scientific, USA). Prior to DNA sequencing, a native barcoding kit (SQK.NBD.114.24) was utilized for library preparation, and the sequencing was conducted on the Oxford Nanopore MinION™ Mk1C platform using the FLO-MIN114 (R10.4.1) flow cell. The MINKNOW program (version 1.11.5) was employed for data collection. The Guppy (v.6.3.2), in high accuracy mode, was utilized for basecalling the MinION™ Mk1C sequencing data (FAST5 files) to generate pass reads (FASTQ format) with a mean quality score > 9. The adaptor and barcode sequences were removed via Porechop (v.0.2.4) [35], and low-quality bases (q < 10) were removed with Nanofilt [36]. The de novo assembly workflow was employed to construct the quality-trimmed reads into contigs using Flye (v.2.9.4) [37]. Further, genome polishing was done by Racon (v.1.5.0) [38]. The Type (Strain) Genome Server (TYGS) was employed to evaluate the species [39]. The potential contamination within the genome and its completeness was evaluated using ConEst16S (https://www.ezbiocloud.net/tools/contest16s) [40], CheckM (v1.2.4) [41], QUAST (v.5.2.0) [42], and BUSCO (v.6.0.0) [43]. The average nucleotide identity (ANI) was assessed using the OrthoANI tool available on the Ezbiocloud server (https://www.ezbiocloud.net/tools/orthoaniu) [44].

### Genome annotation, mapping, subsystem and metabolic pathway analysis

The structural and functional annotation of the assembled genomes were performed by Prokka (v. 1.14.6) [45], RAST (v.1.3) [46], and the Prokaryotic Genome Annotation Pipeline (PGAP) of NCBI (v.6.10) [47]. The annotated genome map was illustrated using Proksee server [48]. Subsystem and functional metabolic pathway analysis was performed using Patric [49] and RAST [46] servers. The biosynthetic gene clusters were determined by using the antiSMASH server [50].

### Antimicrobial resistance gene, virulence genes, mobile genetic elements, and prophase analysis

Within the assembled genomes, the potential antimicrobial resistance genes were evaluated using the Comprehensive Antibiotic Resistance Database (CARD) server [51]. Afterwards, mobile genetic elements and virulence genes were assessed by mobileOG-db [52] and Victors [53] tools, respectively. The Phage integration within the assembled genomes was evaluated through the PHAge Search Tool Enhanced Release (PHASTER) [54,55] and the PHAge Search Tool with Enhanced Sequence Translation (PHATEST) [56] tools.

### Pangenome analysis and pathogenicity profiling

A comprehensive pangenome assessment and comparative genomic analysis were performed to elucidate the intrinsic diversity of the target strain. The objective was to identify unique presence-absence variations (PAVs) in specific genes or gene families among strains from various geographical regions and sources. Ten whole-genome sequences of *V. parahaemolyticus* strains from human sources in different countries and thirteen sequences from non-human sources were obtained from the Bacterial and Viral Bioinformatics Resource Center (BV-BRC) (S1 Data). Analyses included a total of twenty-seven *V. parahaemolyticus* genomes, incorporating the test isolates *V. parahaemolyticus* SU37A and SU91A, using Roary [57], which efficiently constructs large-scale pangenomes and distinguishes core and accessory genes. Pathogenicity profiling and prediction for all isolates with respect to human hosts were conducted using the PathogenFinder (v2.0) web tool from the Center for Genomic Epidemiology, with subsequent data analysis and visualization performed using the Tidyverse package in RStudio [58,59]. The server utilizes four neural network models (Models 1–4), each trained on separate subsets of the original dataset. A prediction score exceeding 0.5 indicates pathogenic potential, while a score below this threshold suggests a lack of pathogenic potential [58,59].

## Results

### Multidrug resistance profile

The presumptive *V. parahaemolyticus* isolate, SU37A, demonstrated total resistance to erythromycin, ceftazidime, nitrofurantoin, meropenem, azithromycin, and ampicillin. This strain also showed an intermediate phenotype to nalidixic acid and cefotaxime. Nonetheless, it remained susceptible to gentamicin, ciprofloxacin, chloramphenicol, amikacin, imipenem, and levofloxacin (S1 Table). Additionally, the isolate SU91A exhibited a resistance phenotype to erythromycin, ceftazidime, nitrofurantoin, meropenem, azithromycin, ampicillin, and cefotaxime. This strain showed an intermediate phenotype to ciprofloxacin and nalidixic acid, yet remained sensitive to gentamicin, chloramphenicol, amikacin, imipenem, and levofloxacin. According to the resistance profiles observed, both strains were identified as MDR, as they showed non-susceptibility to at least one agent across three or more antimicrobial categories (S1 Table).

### Whole genome sequence analysis and functional annotation

Subsequent genome analysis of the isolates SU37A and SU91A revealed that both isolates belong to the *V. parahaemolyticus* as evidenced by the TYGS identification. The findings were validated by the significant ANI scores surpassing the threshold values (>96%), while comparing with the genome of reference strains (S2 Table). Subsequently, the isolates 37A and 91A were classified as “*Vibrio parahaemolyticus* strain SU37A” and “*Vibrio parahaemolyticus* strain SU91A,” respectively. Further, the genome sequences of both isolates have been submitted to GenBank at National Center for Biotechnology Information (NCBI), assigned the accession numbers JBQRUG000000000.1 and JBQWLY000000000.1. The genome of *V. parahaemolyticus* strain SU37A had a coarse and fine consistency of 99.9% and 98.7%, while the *V. parahaemolyticus* strain SU91A had 99.7% and 91.9%, respectively. The completeness of the *V. parahaemolyticus* strain SU37A and *V. parahaemolyticus* strain SU91A was 97.04% and 86.55%, respectively. At the same time, both genomes had the lowest contamination, 1.08% in strain SU37A and 0.84% in strain SU91A. The genome size and GC content of the strains SU37A and SU91A corresponded closely with the reference genomes of *V. parahaemolyticus* (S2 Table).

Following *de novo* genome assembly and annotation, the coding sequence (CDS) ratios of *V. parahaemolyticus* strain SU37A and SU91A were predicted to be 0.929 and 1.012, respectively. The strains also exhibited notable functional and evolutionary diversity, as indicated by the significant number of hypothetical proteins (≥30.0%) identified in the genomes (S2 Table).

**Fig 1:**
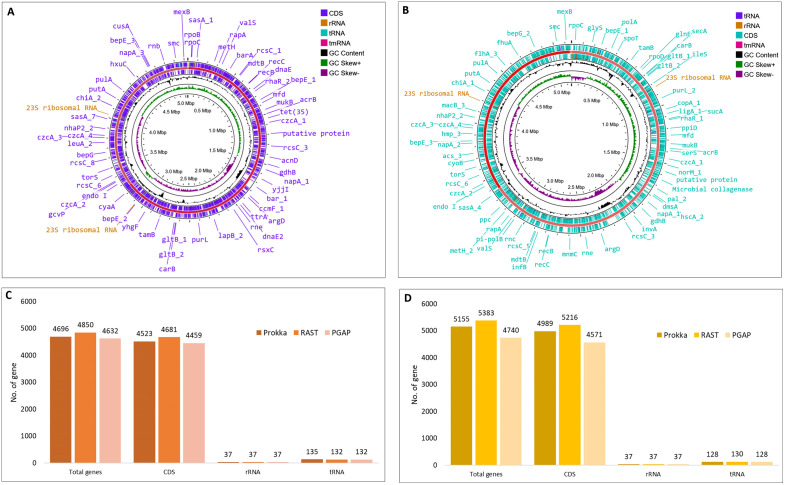
Genome features analysis of the genome SU37A and SU91A. (A). Genome mapping of the *V. parahaemolyticus* strain SU37A featuring CDS, rRNA, tRNA, tmRNA, and GC content. (B) Genome mapping of the *V. parahaemolyticus* strain SU91A featuring CDS, rRNA, tRNA, tmRNA, and GC content. (C) Genome annotation features of the *V. parahaemolyticus* strain SU37A by Prokka, RAST, and PGAP. (D) Genome annotation features of the *V. parahaemolyticus* strain SU91A by Prokka, RAST, and PGAP.

The genome annotation and mapping of these isolates highlighted important genomic features, as illustrated in Fig 1 and detailed in S2 Table. In the genome of *V. parahaemolyticus* strain SU37A, the total number of genes predicted by Prokka, RAST, and PGAP were 4696, 4850, and 4632, respectively. Among these, the number of coding DNA sequences (CDSs) identified were 4523 by Prokka, 4681 by RAST, and 4459 by PGAP. Additionally, all three annotation tools consistently predicted 37 rRNA genes, while the number of tRNA genes was 135 in Prokka and 132 in both RAST and PGAP (Fig 1A, 1C). The genome of *V. parahaemolyticus* strain SU91A has a total of 5155, 5383, and 4740 predicted genes as identified by Prokka, RAST, and PGAP, respectively. The obtained CDSs were 4989 by Prokka, 5216 by RAST, and 4571 by PGAP. Furthermore, all three annotation tools evenly identified 37 rRNA genes, but the number of tRNA genes was 130 in RAST and 128 in both Prokka and PGAP (Fig 1B, 1D).

### Antimicrobial resistance genes, and mobile genetic elements analysis

**Fig 2:**
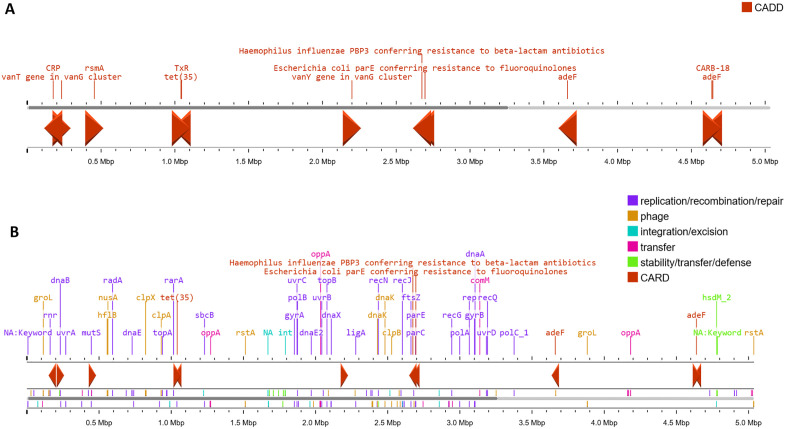
The antibiotic resistance genes and mobile genetic elements found in the genome of *V. parahaemolyticus* strain SU37A. (A) The antibiotic resistance genes cluster predicted by the CARD (*vanT*, *vanY*, *tet*, *parE*, *PBP3*, *adeF*, and *CARB-18*). (B) The locations of mobile genetic elements proximate to the antibiotic resistance genes.

**Fig 3:**
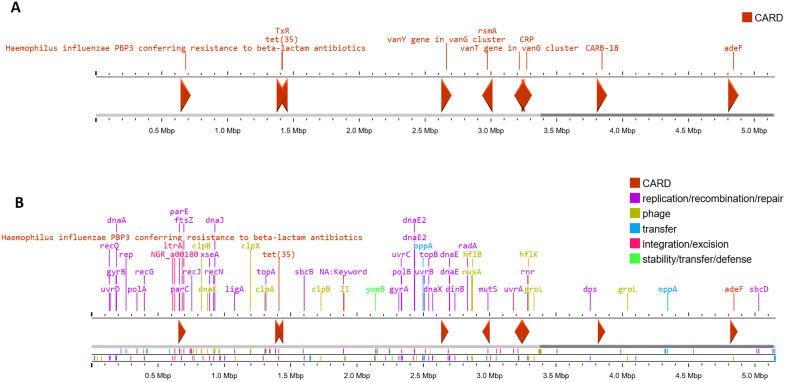
The antibiotic resistance genes and mobile genetic elements found in the genome of *V. parahaemolyticus* strain SU91A. (A) The antibiotic resistance genes cluster predicted by the CARD (*vanT*, *vanY*, *tet*, *parE*, *PBP3*, *adeF*, and *CARB-18*). (B) The locations of mobile genetic elements proximate to the antibiotic resistance genes.

The analysis of antimicrobial resistance genes in the *V. parahaemolyticus* strain SU37A and SU91A revealed an array of resistance factors across antibiotic groups. Both strains contained the *tet* (35) and *adeF* genes, responsible for resistance to several classes of antibiotics, including macrolides, fluoroquinolones, penams, and tetracyclines. Genes responsible for beta-lactam antibiotics resistance were also identified in both strains, including *CARB*-18 and *PBP*s (S3 Table, Fig 2A and 3A). The genomes of the strains also contained *vanY* and *vanT* genes in the *vanG* cluster, which are responsible for vancomycin resistance. Several regulatory genes associated with antibiotic resistance were also identified, including *CRP* and *rsmA* (S3 Table, Fig 2A and 3A). However, the genome of strain SU37A contains a variation in the *parE* gene, which is identical to the *Escherichia coli parE* gene and confers fluoroquinolone resistance; this variation was not observed in SU91A (S3 Table, Fig 2A and 3A). Additionally, the genomes were predicted to contain MGE-associated genes, including those involved in phage functions, recombination and repair, integration and excision, and DNA transfer. Several antimicrobial resistance genes are located in close proximity to these MGE-associated regions. The *PBP3* and the *tet* are positioned adjacent to recombination and integration or excision genes, whereas the efflux-associated resistance gene *adeF* is found near transfer-related elements (Fig 2B and 3B).

### Virulence associated gene analysis

**Fig 4:**
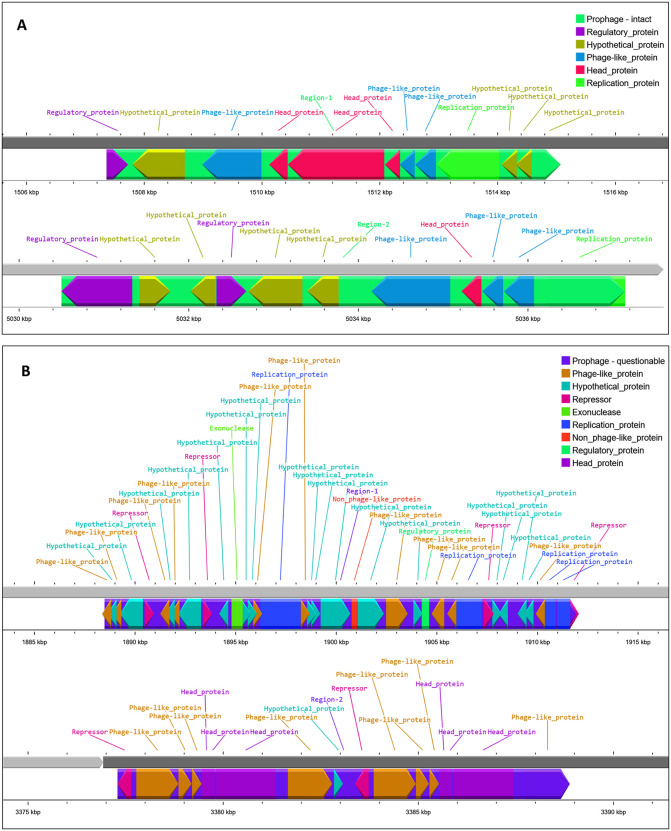
The phage and phage related elements within the genomes of *V. parahaemolyticus* SU37A and SU91A. (A) The phage integrated elements including the gene responsible for synthesis of prophage, phage head, regulatory and replication protein in the genome of strain SU37A. (B) The phage integrated elements including the gene responsible for synthesis of prophage, repressor, exonuclease, phage head, regulatory and replication protein in the genome of strain SU91A.

More than 60 virulence-associated genes were identified in *V. parahaemolyticus* strains SU37A and SU91A. Regulatory and stress response genes, including *rpoS, rpoE, crp, hfq*, and *arcA*, were present in both strains. Genes related to secretion and adhesion, such as *ascV, tolB, tufa*, and *trxA*, were also identified. Both strains have been reported to possess iron-sequestering genes, including *fur, fbp*, and *ccmF*. In addition, metabolism-related genes, such as *aceF, pta, zwf*, and *fabG*, were found in the genomes of SU37A and SU91A. DNA repair genes, including *ruvB* and *recO*, were also identified and contribute to maintaining genomic integrity in response to oxidative and host-induced DNA damage. Nucleotide biosynthesis genes, such as *purF, carA*, and *guaA*, were commonly found in both strains. Energy metabolism genes, including *frdC, atpA*, and *sucA*, support anaerobic respiration and energy conservation in low-oxygen environments (S2 Data). The strains also contain redox homeostasis genes, such as *gshB* and *parE*. Furthermore, membrane stability genes, including *tolB* and *VC2206*, were commonly identified and are involved in maintaining cell membrane integrity and defending against immunological attack. Notably, neither of the strains harbored the two key pathogenic markers, *tdh* and *trh*. The strains were screened for phage and phage-related elements, including phage regulatory and phage-like protein-coding genes. Genes associated with phage head formation, regulatory functions, and replication-related protein synthesis were identified in both SU37A and SU91A. Furthermore, the SU91A genome contained genes encoding repressor and exonuclease proteins (Fig 4).

### Biosynthetic gene cluster and metabolic pathways investigation

**Fig 5:**
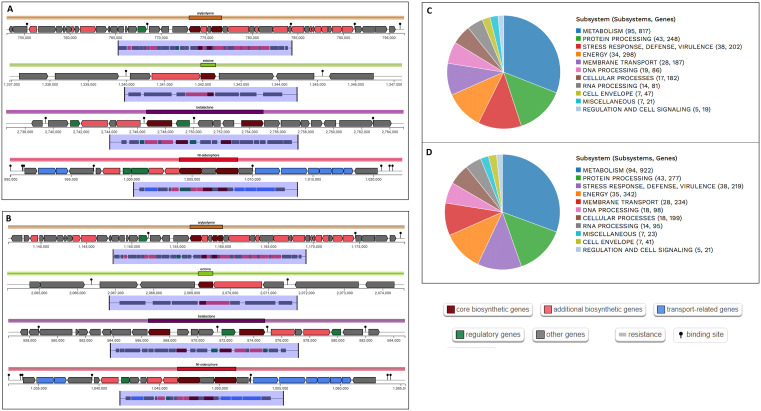
The biosynthetic gene cluster and subsystem analysis of the *V. parahaemolyticus* strains SU37A and SU91A. (A, B) The biosynthetic gene clusters analysis of the strains SU37A and SU91A, including four core (arylpolyene, ectoine, betalactone, NI-siderophore), and other biosynthetic genes. (C, D) The subsystem analysis of the strains SU37A and SU91A, including metabolism, protein processing, stress response, defense, virulence, respiration, membrane transport, cellular processes, regulation, and cell signaling.

The genomes of *V. parahaemolyticus* strains (SU37A and SU91A) contain several major biosynthetic gene clusters, including four core biosynthetic genes, transport-related genes, regulatory genes, and additional associated genes (Fig 5A, 5B). The four primary biosynthetic gene clusters encode NI-siderophore, betalactone, ectoine, and arylpolyene biosynthetic pathways. These biosynthetic gene clusters, which are linked to the production of secondary metabolites, are conserved in both *V. parahaemolyticus* strains SU37A and SU91A.

Subsystem analysis indicated that the strains contained genes associated with metabolism, protein processing, stress response, defense, virulence, respiration, membrane transport, cellular processes, regulation, and cell signaling. Subsequent metabolic pathway analysis identified the involvement of key metabolic processes, including glucose metabolism, amino acid metabolism, glycan biosynthesis and metabolism, and signal transduction. Additional pathways, including lipid metabolism, energy metabolism, polyketide biosynthesis, and secondary metabolite biosynthesis, were also present in the strains. The strains also harbored pathways involved in the biodegradation and metabolism of xenobiotics (Fig 5C, 5D; S3 Data). The strains had multiple routes for secondary metabolite biosynthesis, including those for carotenoid, zeatin, phenylpropanoid, flavonoid, and novobiocin production. The identification of xenobiotic degradation pathways, including those for 2,4-dichlorobenzoate, toluene, xylene, geraniol, ethylbenzene, trinitrotoluene, styrene, atrazine, and caprolactam (Fig 5C, 5D, S3 Data), demonstrates the strain’s capacity to withstand and adapt to adverse environmental conditions.

### Pangenome analysis and genome-wide pathogenicity profiling

**Fig 6:**
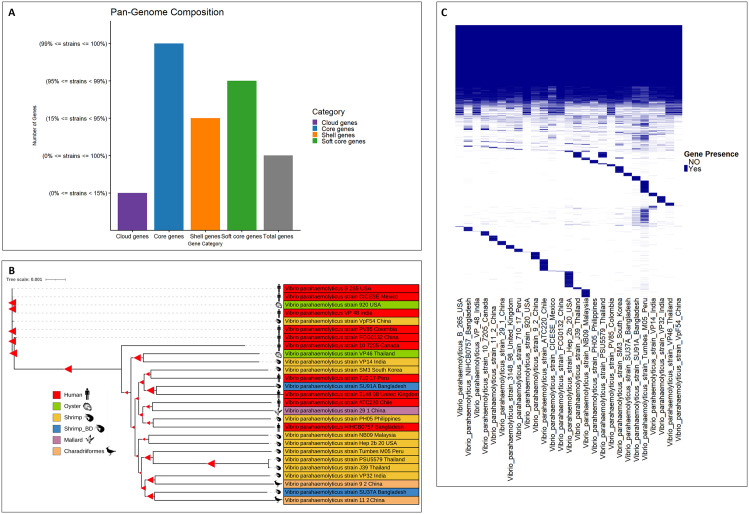
Pangenome analysis of the *V. parahaemolyticus* across different geographics and origins. (A) The genome comparison of the 27 different genomes reveled different genomic composition including core (blue), soft (green), shell (yellow) and cloud (purple) genes across the strains. (B) The phylogenetic analysis of the 27 pan-genome depicts the evolutionary relationship of the strains of different geographics and origins (Isolates from different sources are depicted in different color codes, red-human, green-oyster, yellow-shrimp, blue- shrimp_Bangladesh, pink-mallard, and orange- Charadriiformes). (C) Genome alignment of the strains suggests significant genes conservation across the strains (Blue and color denotes the presence and absence of genes, respectively).

A comprehensive pangenome analysis was conducted using 27 whole genome sequences of *V. parahaemolyticus* isolates from diverse geographic locations and sources, including strains SU37A and SU91A from the present study. The analysis identified a core genome of 3,590 genes, along with 245 soft core genes, 1,159 shell genes, and 4,537 cloud genes. In total, 9,531 genes were identified and analyzed within the pangenome (Fig 6A). The pangenome-wide study demonstrated that *V. parahaemolyticus* strains from non-human sources showed significant genomic and phylogenetic resemblance to clinical isolates from human disease. The *V. parahaemolyticus* strain SU91A, acquired from Bangladesh, was very closely related to the clinical isolate *V. parahaemolyticus* strain 3148−98 from the United Kingdom. Conversely, the *V. parahaemolyticus* strain SU37A, another strain of this study, exhibited a close phylogenetic relationship to the non-clinical isolate *V. parahaemolyticus* strain 11−2 from China, which originated from Charadriiformes (Fig 6B, 6C). Additionally, *V. parahaemolyticus* strain 710−17 from shrimp in Peru and *V. parahaemolyticus* strain SM3, a clinical isolate from South Korea, were identified as belonging to the same clade. Meanwhile, the clinical isolate *V. parahaemolyticus* strain NIHCB0757 in Bangladesh, and the strain PH05 from shrimp in the Philippines, exhibited significant genomic features and a proximate phylogenetic relationship, as both were categorized within the same clade with minimal evolutionary divergence. Moreover, the *V. parahaemolyticus* strain 29−1 from mallards in China showed a close phylogenetic relationship with the clinical strain ATC220 in Chile. Similar trends were observed for other isolates. High levels of phylogenetic closeness were observed in the strain VP46 from oysters in Thailand and the strain VP14 from shrimp in India. Similarly, the strain 9−2 from Charadriiformes in China exhibited a close genetic relationship to the shrimp-associated strain VP32 in India (Fig 6B, 6C). Both strains were concentrated within the same clade and exhibited minimal evolutionary distance.

**Fig 7:**
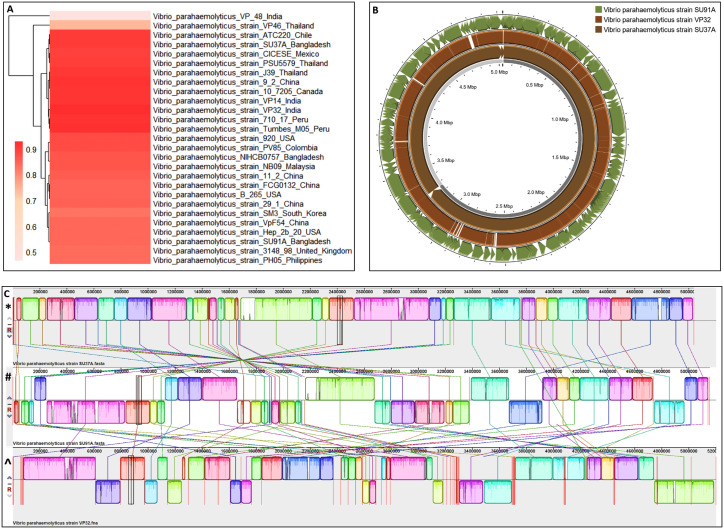
Comparative genome analysis of the *V. parahaemolyticus* strains across different geographics and origins. (A) Pathogenicity scores of the *V. parahaemolyticus* strains, with color gradient depicting the degree of pathogenicity (white to red, from lower to higher pathogenicity). (B) Circos plot of the most pathogenic human strains VP32 and the strain SU37A and SU91A. (C) Syntenic genomic comparison of the human *V. parahaemolyticus* strain VP32 (^) with strains SU37A (*) and SU91A (#). Colored aligned blocks indicate highly conserved genomic regions shared among the strains.

The genome-wide pathogenicity analysis of 27 *V. parahaemolyticus* isolates provided significant insights into the species’ pathogenic potential. All the strains, regardless of their human or non-human origin, were predicted to be pathogenic, including the *V. parahaemolyticus* strains SU37A and SU91A (Fig 7). The observed genomic and phylogenetic relatedness across the isolates, along with consistently high pathogenicity scores, highlights the widespread virulence potential of *V. parahaemolyticus* across diverse ecological environments. The *V. parahaemolyticus* strain VP32, isolated from shrimp in India, demonstrated the highest pathogenic potential among all assessed isolates, with a pathogenicity score of 0.927 (Fig 7). The predicted pathogenicity scores for strains SU37A and SU91A were 0.904 and 0.840, respectively. Fig 7 presents a comparative genomic overview of these strains relative to VP32. Other isolates derived from shrimp, such as *V. parahaemolyticus* strains Tumbes M05, VP14, and J39 from Peru, India, and Thailand, respectively, also exhibited significant pathogenic potential. Meanwhile, two clinical strains, 710−17 from Peru and 10−7205 from Canada, were predicted to have higher pathogenicity. Furthermore, the strain 9−2 from Charadriiformes in China had a similar pathogenic profile.

## Discussion

The aquaculture sector in Bangladesh has experienced rapid expansion, however, has been accompanied by a rise in infectious diseases, particularly those caused by *Vibrio* species that are prevalent in estuarine and coastal shrimp-farming regions [3]. These pathogens adversely affect shrimp health, reduce productivity, diminish export quality, and pose risks to human food safety, thereby threatening the sustainability of aquaculture [5]. Whole genome analysis offers a comprehensive method for investigating *Vibrio* epidemiology, virulence factors, antimicrobial resistance, mobile genetic elements, and horizontal gene transfer, providing greater resolution than traditional phenotypic or single-gene approaches [19]. Comparative genomics facilitates strain-level discrimination, source tracking, and evolutionary analysis, thereby enhancing early outbreak detection, risk assessment, and targeted surveillance to safeguard shrimp production and public health [21,22]. Therefore, this study described the genomes of two *V. parahaemolyticus* strains (SU37A and SU91A) sequenced using Oxford Nanopore long-read technology. The sequences were subsequently assembled and annotated using multiple bioinformatics tools. The genomics validation using ANI scores (>96%) revealed that the isolates belong to the genus *V. parahaemolyticus*, as compared with the reference strain genomes. The genome sequence analyses of both strains revealed high genome quality, featured by their coarse consistency, fine consistency, completeness, and contamination. The CDS ratios of the isolates indicate a high-quality genome assembly, as they fall within the standard CDS ratio range (0.8–1.2) generally accepted by the NCBI for well-assembled bacterial genomes. The strains also exhibited notable functional and evolutionary diversity, as indicated by the significant number of hypothetical proteins (≥30.0%) identified in the genomes. Comprehensive analysis of hypothetical proteins in both strains may facilitate the discovery of previously unrecognized biological functions and enable the identification of novel diagnostic biomarkers or therapeutic targets for *Vibrio* species [60]. Subsequent research should prioritize the functional characterization of these proteins through advanced computational approaches, including homology modeling, protein–protein interaction analysis, and machine learning techniques [61,62].

Both strains harbored the *tet(35)* gene, which is associated with tetracycline resistance mediated by ATP-binding cassette (ABC) efflux pumps, and is likely to contribute to reduced tetracycline susceptibility [63]. Beta-lactam antibiotic resistance was also forecasted in both strains, attributable to the presence of *CARB-18* and *PBP*s genes. The *PBP*s gene contributes to resistance phenotypes against cephalosporins, and penams through mutations in PBPs. The S385T substitution in PBP3 could potentially reduce the protein’s affinity for beta-lactams and may be associated with altered susceptibility, although its direct contribution to antimicrobial resistance of *Vibrio* remains to be fully established [64]. Consequently, these mechanisms may enable *V. parahaemolyticus* strains SU37A and SU91A to preserve cell wall integrity during antibiotic exposure, which could contribute to reduced susceptibility to beta-lactam antibiotics [65]. The genomes of the strains SU37A and SU91A also contained *vanY* and *vanT* genes in the *vanG* cluster, which are responsible for vancomycin resistance via modifications in cell wall targets [66]. However, the prediction of these genes in *V. parahaemolyticus* lacks direct therapeutic or diagnostic relevance because vancomycin is ineffective against Gram-negative bacteria [67]. Consequently, the importance of these genes is primarily ecological and evolutionary, indicating the presence of environmental gene reservoirs rather than representing clinically actionable antimicrobial resistance. The strains harbored regulatory genes (*CRP* and *adeF*) that may be associated with resistance to multiple antibiotic classes, including macrolides, fluoroquinolones, penams, and tetracyclines [68,69]. These strains may also exhibit multidrug resistance due to the presence of the regulatory *rsmA* gene, which is associated with the RND efflux pump and has been reported to affect susceptibility to fluoroquinolones, diaminopyrimidines, and phenicol [70]. However, the genome of strain SU37A harbors a variation in the *parE* gene, which is associated with fluoroquinolone resistance, whereas this variation is absent in strain SU91A [71]. This genetic variation results in a point mutation (D476N) in the ParE protein, which may induce fluoroquinolone resistance by altering the structure of the DNA gyrase subunit. These structural modifications may inhibit the strain SU37A’s ability to bind its target, thereby enhancing bacterial survival in the presence of fluoroquinolones [72]. In both strains (SU37A and SU91A), several antimicrobial resistance determinants were identified in close proximity to MGE-associated regions. The *PBP3* and *tet* genes were positioned adjacent to recombination and integration/excision modules, while the efflux-associated resistance gene *adeF* was found near transfer-related elements. This genomic organization indicates that these resistance genes are likely associated with horizontally acquired regions and may be mobilized via MGE-mediated gene transfer [73].

The strains had several virulence genes, which are essential for modulating virulence and facilitating the adaptation of *Vibrio* species to diverse and stressful environments, such as nutritional scarcity and host immune responses. The *rpoS* and *rpoE* genes encode sigma factors that regulate stress responses and pathogenicity by enhancing bacterial survival during nutritional limitation, oxidative damage, and envelope stress [74]. The *hfq* gene encodes an RNA chaperone that mediates small RNA regulation of stress, motility, and quorum sensing [75]. Notably, *arcA* genes of the ArcAB system responds to redox fluctuations, enhancing energy metabolism and regulating motility, biofilm development, and virulence under low-oxygen conditions [76]. Genes related to secretion and adhesion, which are critical for host colonization and toxin delivery. The *ascV* gene, a component of the Type III Secretion System (T3SS), is involved in the translocation of effector proteins into host cells, facilitating cytotoxicity and immune evasion [77]. The *tolB* gene contributes to outer membrane stability and maintains cell envelope integrity, which is important for adhesion and virulence [78]. The *tufa* gene encodes elongation factor Tu, which is involved in protein synthesis and also functions as an adhesin to promote host cell attachment [79]. Both strains have been reported to possess iron-sequestering genes, including *fur, fbp*, and *ccmF*. Effective colonization and pathogenicity in these species depend on multiple functional gene clusters that support growth, defense, and adaptability within the host. The identified iron-sequestering genes regulate iron homeostasis, which is essential for bacterial metabolism and survival in iron-deficient host environments [80]. In addition, presence of metabolism-related genes (*aceF, pta, zwf*, and *fabG*) may enhance the strains’ virulence by optimizing central carbon metabolism, fatty acid biosynthesis, and energy generation, all of which are critical for toxin release and motility [81]. Energy metabolism genes, including *frdC, atpA*, and *sucA*, support anaerobic respiration and energy conservation in low-oxygen environments. The strains also contain redox homeostasis genes, such as *gshB*, which protect against oxidative stress by producing glutathione and activating peroxiredoxins [82]. Furthermore, membrane stability genes, including *tolB* and *VC2206*, were commonly identified and are involved in maintaining cell membrane integrity and defending against immunological attack [78]. However, the absence of *tdh* and *trh* in strains SU37A and SU91A suggests these isolates likely belong to the environmental lineage and have a reduced capacity to cause acute gastroenteritis. However, the absence of such virulence markers does not mean these bacteria are entirely non-pathogenic [12,13]. Other virulence mechanisms, such as Type III secretion systems, adhesion factors, biofilm formation, iron acquisition, and stress adaptation, may still support colonization, persistence, and opportunistic infection [77]. These strains may therefore retain ecological fitness and limited pathogenic potential without the classical hemolysin markers.

Within the genomes, the predicted four primary biosynthetic gene clusters are recognized for facilitating adaptation to environmental conditions and may contribute to the pathogenicity and ecological viability of these strains. Arylpolyene biosynthetic gene clusters encode the production of carotenoid-like polyene pigments that enhance biofilm structural integrity and confer protection against oxidative stress. The detection of arylpolyene clusters in the genomes of both strains indicates a potential role in oxidative resilience and persistence, which are critical for infection establishment and sustained colonization of host environments [83,84]. Among the core biosynthetic gene clusters, the NI-siderophore represents the most evident factor influencing the bacterium’s virulence. A key virulence strategy of pathogenic *Vibrio* species is iron acquisition in iron-limited host environments through siderophores, such as vibrioferrin [85]. The presence of NI-siderophore clusters enables *V. parahaemolyticus* to efficiently extract iron from the host environment, facilitating growth and colonization during infection. Also, siderophore-mediated iron acquisition has been linked to increased biofilm formation and tolerance to oxidative stress. The presence of these gene clusters in both SU37A and SU91A is predicted to contribute to traits including biofilm formation and oxidative stress tolerance. Nevertheless, these associations are currently supported only by genomic predictions and require experimental validation. The identification of xenobiotic degradation pathways, including those for 2,4-dichlorobenzoate, toluene, xylene, geraniol, ethylbenzene, trinitrotoluene, styrene, atrazine, and caprolactam, demonstrates the strain’s capacity to withstand and adapt to adverse environmental conditions. This metabolic flexibility and resilience are further supported by the combined metabolic capabilities of strains SU37A and SU91A, which play a critical role in their survival, metabolic activity, virulence, and pathogenicity.

The findings of pangenome analysis indicate that the *V. parahaemolyticus* pangenome is likely expanding and demonstrates substantial genetic variation among the analyzed strains, which reflects the species’ genomic diversity. This variation may results from mechanisms such as horizontal gene transfer, mutation, and adaptation to diverse ecological niches [86]. Analysis of strains SU37A and SU91A reveals potential genomic features that may enhance adaptability and identifies genes or variations not previously reported in earlier genome analyses. Phylogenetic analysis showed that several *V. parahaemolyticus* strains derived from non-human sources shared genomic similarity with clinical isolates associated with human disease. In particular, strain SU91A clustered closely with the clinical isolate *V. parahaemolyticus* strain 3148−98, whereas strain SU37A showed a closer relationship with the non-clinical isolate strain 11−2. The presence of strains from different hosts and geographic regions within the same clade may indicate possible shared evolutionary backgrounds among certain *V. parahaemolyticus* lineages. Predicted pathogenicity scores for strains SU37A and SU91A were 0.904 and 0.840, respectively, suggesting a potential capacity for virulence. However, further experimental validation would be required to confirm the pathogenic potential of these strains. Overall, the observed genomic and phylogenetic relationships highlight the genetic diversity of *V. parahaemolyticus* and suggest that strains from diverse ecological sources may harbor genomic features associated with pathogenicity.

## Conclusion

The comprehensive genomic analysis of *V. parahaemolyticus* strains SU37A and SU91A revealed several significant features related to their antibiotic resistance and virulent traits. The identification of multidrug resistance-associated efflux pumps and β-lactamase genes underscores the clinical significance of these strains. The presence of mobile genetic elements adjacent to resistance genes may indicate a potential for horizontal gene transfer and dissemination of resistance traits. Virulence profiling identified genes related to regulatory mechanisms, stress responses, secretion systems, adhesion, and iron acquisition, indicating the strains’ ability to persist under adverse conditions and evade host immune defenses. Comparative and pangenome analyses demonstrated considerable genetic diversity, substantial evolutionary adaptation, and gene exchange among strains from varied ecological and host backgrounds. Phylogenomic clustering revealed strong associations between clinical and environmental strains from geographically distinct regions. The observed genetic and metabolic versatility of these strains emphasize the growing public health risk posed by *V. parahaemolyticus* in both aquatic and clinical settings. Although multiple antibiotic resistance and virulence genes were predicted from the genome, these findings were not validated by phenotypic assays. Furthermore, the study remains primarily descriptive, and functional experiments are required to determine the biological significance of the identified genomic features. Sampling was also restricted to a single location, which may not adequately capture the broader geographic diversity of *V. parahaemolyticus* associated with shrimp.

## Supporting information

S1 Table Interpretative criteria used to determine antimicrobial susceptibility with the disk diffusion test in the *V. parahaemolyticus* strain SU37A and SU91A. (DOCX)

S2 Table The genomic features of the *V. parahaemolyticus* strain SU37A and SU91A. (DOCX)

S3 Table List of antibiotic resistance genes of the *V. parahaemolyticus* strain SU37A and SU91A along their classification, resistance mechanism and AMR gene family. (DOCX)

S1 Data Pangenome analysis. (XLSX)

S2 Data Virulence associated genes of the SU37A and SU91A. (XLSX)

S3 Data Metabolic pathway analysis of SU37A and SU91A. (XLSX)
