# Supplementary material for: Whole genome and phylogenomic insights into Vibrio parahaemolyticus from Pacific White Shrimp reveal resistance and virulence traits in Bangladeshi aquaculture
Source: PLoS One. 2026 Apr 9;21(4):e0346962. doi: 10.1371/journal.pone.0346962 (PMC13065059; doi:10.1371/journal.pone.0346962)
Supplement: S1 Table — (DOCX) [file pone.0346962.s001.docx]

**Table S1.** Interpretative criteria used to determine antimicrobial susceptibility with the disk diffusion test in the *V. parahaemolyticus* strain SU37A and SU91A.

| **Antibiotics** | **Antibiotic class** | **Zone diameter interpretative criteria (mm)** | | | **Protocol** | **Phenotypic profile** | |
| --- | --- | --- | --- | --- | --- | --- | --- |
|  |  | **Susceptible** | **Intermediate** | **Resistant** |  | Strain SU37A | Strain SU91A |
| Erythromycin | Macrolide | ≥12 | - | ≤12 | EUCAST interpretive criteria for *Vibrio* species | Resistant | Resistant |
| Gentamicin | Aminoglycoside | ≥15 | 13-14 | ≤12 | CLSI M45 interpretive criteria for *Vibrio* species | Sensitive | Sensitive |
| Ceftazidime | Extended-spectrum cephalosporin | ≥22 | - | ≤22 | EUCAST interpretive criteria for *Vibrio* species | Resistant | Resistant |
| Ciprofloxacin | Fluoroquinolone | ≥21 | 16-20 | ≤15 | CLSI M45 interpretive criteria for *Vibrio* species | Sensitive | Intermediate |
| Nalidixic Acid | Quinolone | ≥19 | 14-18 | ≤13 | CLSI M100 interpretative criteria for Enterobacterales | Intermediate | Intermediate |
| Chloramphenicol | Phenicol | ≥18 | 13-17 | ≤12 | CLSI M45 interpretive criteria for *Vibrio* species | Sensitive | Sensitive |
| Nitrofurantoin | Nitrofuran | ≥17 | 15-16 | ≤14 | CLSI M100 interpretative criteria for Enterobacterales | Resistant | Resistant |
| Meropenem | Carbapenem | ≥24 | - | ≤24 | EUCAST interpretive criteria for *Vibrio* species | Resistant | Resistant |
| Amikacin | Aminoglycoside | ≥17 | 15-16 | ≤14 | CLSI M45 interpretive criteria for *Vibrio* species | Sensitive | Sensitive |
| Azithromycin | Macrolide | ≥16 | - | ≤16 | EUCAST interpretive criteria for *Vibrio* species | Resistant | Resistant |
| Ampicillin | Penicillin | ≥17 | 14-16 | ≤14 | CLSI M45 interpretive criteria for *Vibrio* species | Resistant | Resistant |
| Imipenem | Carbapenem | ≥23 | 20-22 | ≤19 | CLSI M45 interpretive criteria for *Vibrio* species | Sensitive | Sensitive |
| Cefotaxime | Extended-spectrum cephalosporin | ≥26 | 23-25 | ≤22 | CLSI M45 interpretive criteria for *Vibrio* species | Intermediate | Resistant |
| Levofloxacin | Fluoroquinolone | ≥23 | - | ≤23 | EUCAST interpretive criteria for *Vibrio* species | Sensitive | Sensitive |
