# Supplementary material for: Whole genome and phylogenomic insights into Vibrio parahaemolyticus from Pacific White Shrimp reveal resistance and virulence traits in Bangladeshi aquaculture
Source: PLoS One. 2026 Apr 9;21(4):e0346962. doi: 10.1371/journal.pone.0346962 (PMC13065059; doi:10.1371/journal.pone.0346962)
Supplement: S2 Table — (DOCX) [file pone.0346962.s002.docx]

| Features | Strain | |
| --- | --- | --- |
|  | **SU37A** | **SU91A** |
| Coarse consistency (%) | 99.9 | 99.7 |
| Fine consistency (%) | 98.7 | 91.9 |
| Completeness (%) | 97.04 | 86.55 |
| Contamination (%) | 1.08 | 0.84 |
| GC content (%) | 45.43 | 45.34 |
| Contig count | 3 | 3 |
| DNA size (bp) | 5037599 | 5156510 |
| Contigs N50 (bp) | 3252908 | 3376922 |
| Contigs L50 | 1 | 1 |
| ANI score (%) | 98.49 | 98.44 |
| Protein-Encoding Genes with Functional Assignment | 3238 | 3606 |
| Protein-Encoding Genes without Functional Assignment | 1443 | 1610 |
| % Protein-Encoding Feature Coverage | 92.92 | 101.15 |
| % Features that are Hypothetical | 30.83 | 30.87 |
| % Features that are in Local Protein Families | 96.37 | 96.05 |

**Table S2.** The genomic features of the *V. parahaemolyticus* strain SU37A and SU91A.
