# Supplementary material for: Whole genome and phylogenomic insights into Vibrio parahaemolyticus from Pacific White Shrimp reveal resistance and virulence traits in Bangladeshi aquaculture
Source: PLoS One. 2026 Apr 9;21(4):e0346962. doi: 10.1371/journal.pone.0346962 (PMC13065059; doi:10.1371/journal.pone.0346962)
Supplement: S3 Table — (DOCX) [file pone.0346962.s003.docx]

**Table S3.** List of antibiotic resistance genes of the *V. parahaemolyticus* strain SU37A and SU91A along their classification, resistance mechanism and AMR gene family.

| Strain | Gene | SNPs | Drug Class | Resistance Mechanism | AMR Gene Family |
| --- | --- | --- | --- | --- | --- |
| SU37A/SU91A | tet(35) | n/a | tetracycline antibiotic | antibiotic efflux | ATP-binding cassette (ABC) antibiotic efflux pump |
|  | CRP | n/a | macrolide antibiotic; fluoroquinolone antibiotic; penam | antibiotic efflux | resistance-nodulation-cell division (RND) antibiotic efflux pump |
|  | CARB-18 | n/a | penam | antibiotic inactivation | CARB beta-lactamase |
|  | vanY gene in vanG cluster | n/a | glycopeptide antibiotic | antibiotic target alteration | vanY; glycopeptide resistance gene cluster |
|  | adeF | n/a | fluoroquinolone antibiotic; tetracycline antibiotic | antibiotic efflux | resistance-nodulation-cell division (RND) antibiotic efflux pump |
|  | Haemophilus influenzae PBP3 conferring resistance to beta-lactam antibiotics | S385T | cephalosporin; cephamycin; penam | antibiotic target alteration | Penicillin-binding protein mutations conferring resistance to beta-lactam antibiotics |
|  | vanY gene in vanG cluster | n/a | glycopeptide antibiotic | antibiotic target alteration | vanY; glycopeptide resistance gene cluster |
|  | vanT gene in vanG cluster | n/a | glycopeptide antibiotic | antibiotic target alteration | glycopeptide resistance gene cluster; vanT |
|  | rsmA | n/a | fluoroquinolone antibiotic; diaminopyrimidine antibiotic; phenicol antibiotic | antibiotic efflux | resistance-nodulation-cell division (RND) antibiotic efflux pump |
| SU37A | Escherichia coli parE conferring resistance to fluoroquinolones | D476N | fluoroquinolone antibiotic | antibiotic target alteration | fluoroquinolone resistant parE |
